# Cost-benefit analysis for health project evaluation (example of a Russian outpatient clinics' project in the Novosibirsk region)

**DOI:** 10.3389/fpubh.2023.1073964

**Published:** 2023-01-27

**Authors:** Tatyana Novikova, Maria Kaneva, Mokhidilkhon Zafarjonova

**Affiliations:** ^1^Department of Economics, Novosibirsk State University, Novosibirsk, Russia; ^2^Laboratory for Modeling and Analysis of Economic Processes, Institute of Economics and Industrial Engineering Siberian Branch of the Russian Academy of Sciences, Novosibirsk, Russia; ^3^Department for Regional and Municipal Governance, Institute of Economics and Industrial Engineering Siberian Branch of the Russian Academy of Sciences, Novosibirsk, Russia

**Keywords:** health projects, financial and economic efficiency, social effects, cost-benefit analysis, public-private partnership

## Abstract

**Introduction:**

The development of social infrastructure projects in medicine corresponds to transforming public priorities toward social development in general and health care in particular. Therefore, there is a need to develop comprehensive quantitative methods for evaluating such projects.

**Methods:**

This paper uses a combination of two approaches: first, cost-benefit analysis taking into account the relationship between financial and economic efficiency; second, the study of the efficiency of participation in a public-private partnership concerning project efficiency. The model's financial bloc is focused on analyzing the return on investment in fixed and working capital, considering the terminal value. The economic bloc includes social and tax effects (along with environmental, price, indirect, and other specific public effects). We apply fixed effects regression models to calculate multipliers used to estimate the social effects. Multipliers are based on: public health expenditure, human development index, and life expectancy. The proposed methodology has been adapted for evaluating the Seven Polyclinics' project as a flagship project for developing social infrastructure in the Novosibirsk Region.

**Results and discussion:**

The evaluation results revealed a deficient level of financial efficiency of the project characterized by negative net present value and low internal rate of return. Simultaneously, the efficiency of participation in the project for private investors using the public-private partnership mechanism is characterized by high rates of return on private investment. In the transition to the economic analysis, the results fundamentally change, taking into account social and tax effects and detecting an exceptionally high level of all economic indicators of the project. As the project's primary beneficiaries, the economic analysis identified polyclinic patients who received the opportunity to acquire new medical services. At the same time, within the financial analysis framework, the mechanisms for implementing the project were determined, ensuring the consistency of interests. The distribution of effects among the project participants was compared for various funding methods, including the public-private partnerships mechanism. It is shown that the project implementation leads to significant social effects and provides a noticeable improvement in population health. The proposed methodology can be used for decision making on the implementation of similar projects.

## 1. Introduction

Implementing social infrastructure projects has become crucial in forming the institutional foundations of scientific and technological development. To justify and implement such projects successfully, it is essential to develop adequate project analysis tools that consider their specifics.

Social infrastructure encompasses such sectors as healthcare, educational institutions, housing, and communal services, as well as art, culture, and recreation facilities, which are of growing importance in the context of escalating global challenges and a corresponding changing of priorities at the global, national, regional, and municipal levels. In the twentifirst century, investments in infrastructure, especially social infrastructure, have come under the scrutiny of the public and international organizations. Several studies have demonstrated that the existing infrastructure gap in most world countries threatens sustainable economic development. To meet the need, the required additional annual investment in social infrastructure is estimated at around 0.7–1% of GDP in Europe, 1% in the USA, 1.2–1.8% in Japan, and 2.4% in developing Asia (1). The COVID-19 pandemic has highlighted more than ever the chronic underinvestment in social infrastructure due to the surge in healthcare demand, the sudden shift to remote learning, and so on.

Despite changing priorities, the government budgetary and non-budgetary institutions have limited opportunities to solve the underinvestment problem. Hence, it becomes urgent to develop an adequate method for evaluating projects and substantiating the interaction mechanisms of investment stakeholders in this area.

The vital point to consider in the evaluation is that social effects are inherent to social infrastructure projects. These effects come to the fore in the project analysis and thus require a particular assessment approach. Assessment of financial (commercial) efficiency is not enough: the chosen method must allow for simultaneous evaluation of the project's economic (public) efficiency. Health infrastructure development is heavily influenced by the industry-specific features of the relevant projects, representing an important and complex sub-sector of social infrastructure.

In various literature, social effects are defined in different ways. The concept used in this article is closest to Rosenzweig et al. approach: “by [social] impact, we mean the portion of the total outcome that happened as a result of the activity of the venture [or project], above and beyond what would have happened anyway” (2). Here, “above and beyond what would have happened anyway” refers to the difference between the social effects in the situation “with the project” and the social effects in the situation “without the project”. Social effects can be positive and negative, respectively, associated with social benefits and costs. Then the net social effect is measured by the difference between them (3). Social costs (benefits) can be defined as the sum of the private costs (benefits) of individuals or firms carrying out an activity (project, program, production) and the costs(benefits) of third parties outside the activity (project, program, production) (4). The latter are called externalities.

Social impacts, concurrent with economic and environmental ones, are essential for capturing the actual costs and benefits of the intervention (policy, projects) (5). Onyx et al. (6) describe social impacts as “illusive, partly because it does not lend itself readily to a monetary analysis; qualitative rather than quantitative; long term rather than short term; diffuse and multi-layered rather than specific and focused; and probably meaning different things in different contexts”.

In this paper, an investment project in the field of healthcare will be evaluated. The distinctive features of healthcare predetermine the necessity of applying unique methods of project analysis to justify its development. Such practices have been formed within the framework of relevant specific approaches to evaluating public sector projects of the economy, for instance, cost-benefit analysis (CBA).

In this paper, we follow the cost-benefit analysis (CBA) methodology to evaluate a project (policy) consequence in monetary terms for all members of society. The terms “costs” and “benefits” in the name of this method refer to broad sense (7), following the well-known formulation of Mirrlees (8), in which the concepts of social, public, and economic indicators are used as synonyms and are opposed to private and financial indicators. Such practices have been formed within the framework of relevant specific approaches to evaluating projects of the public sector economy.

During the twenteeth century, methods for calculating social benefits in monetary terms were elaborated and used extensively. Initially, they were used to assess national public sector projects, then applied to projects of international financial institutions (both in the private and public sectors) (9). One of the prominent “instrumental” methods for calculating social benefits, developed in the European Guide (10), is the multiplication of private benefits by conversion factors. Over recent years, social indicators, along with environmental ones, have been brought to prominence. The accepted project evaluation standards and even the names of the main methods began to include the specified pair of terms. Then the social benefits and costs are used fairly broadly, combining all social effects except environmental ones. This refers to both universal documents (11, 12) and to the regulations for evaluating healthcare projects with an emphasis on social impact (13).

The cost-benefit analysis is widely applied in Russia. The methodological approach of this direction formed the basis of the Methodological Recommendations (14), which remains the main official guide to project evaluation. In this document, the combination of financial and economic analysis began to be denoted by commercial and public efficiency analysis terms. Moreover, along with the efficiency of the project, the authors proposed to simultaneously consider the efficiency of participation in the project of various agents of investment activity.

Nevertheless, individual authors and official Guidelines of international financial organizations (15, 16) indicate CBA as the most complete and systematic methodological tool for evaluating social infrastructure projects in monetary terms. It does not mean the use of a single method is recommended. Since the middle of the twenteeth century, a multidimensional approach has always been the starting point of project analysis. The considered methods of monetary valuation correspond to the financial and economic aspects, but they are supplemented by environmental, social (without monetary measurements or with their use only for certain types of impacts), marketing, technical, and institutional analysis. Suppose it is possible to monetize the project's impact on one of the above aspects. In that case, the received quantitative results are included in the assessment as the difference between financial and economic analysis.

In CBA, to assess the impact of medical expenses on economic growth, the method of budgetary social multipliers is widely used, especially lately (17–19). It calculates the change in the resultant economic indicators (for example, regional value added) per 1 ruble of growth in healthcare spending. Similar indicators are estimated for other sectors and the economy, while the resultant values differ notably. Estimates of fiscal multipliers in the United States range from 0 to 2, with the most well-known level being 1.5, considering differences between states (19). In a study by the International Monetary Fund, the overall fiscal multiplier in Europe was equal to 1.7, while the multipliers in the health, education, and social protection sectors were estimated at a substantially higher level of 3.0. According to the leading European economists, this figure was 1.6, including 4.3 in healthcare (20). For Asian countries, it is estimated in the range from 0.73 to 0.88 (21).

Based on the above, in this paper, we present an assessment of the project in health care – construction of polyclinics in the Novosibirsk region, Russia. This paper uses a combination of two approaches: first, cost-benefit analysis taking into account the relationship between financial and economic efficiency; second, the study of the efficiency of participation in a public-private partnership in relation to project efficiency. Methods are described in the section that follows.

## 2. Materials and methods

### 2.1. Financial and economic model of health project

The model of an investment project in medicine includes two interconnected blocs. The model's financial bloc is focused on analyzing the financial return on investment in fixed and working capital. To conduct such an analysis and calculate the system of indicators of financial efficiency, we construct the corresponding cash flows. They are determined based on benefits and costs, easily observable in the market interaction process of participants. The related cash flows are presented as equation (1). They are calculated based on traditional methods: for the investment period, capital costs and investments in the growth of working capital are included; for the operational period, the cash inflows from the income after deduction of operating expenses and taxes are included; and for the liquidation period (the period of conditional closing of the project), the terminal (residual) value is taken. To present the considered cash flows in the context of PPP agreements, we divide each of the operating period components into two parts, which correspond to the two parties to the contract: the concessionaire as a private partner with index *c* and the government, which is presented by consolidated budget and clinics as a public partner with index *g*.


(1) $$\begin{array}{l} \begin{array}{lll} CFF_{t} & = & -I_{t}+X_{t}+T_{t}^{VA}-M_{t}-T_{t}+L_{T}=-I_{t}+X_{t}^{c}+X_{t}^{g}+T_{t}^{VA} \end{array}\\ \;\begin{array}{ll} - & M_{t}^{c}-M_{t}^{g}-T_{t}^{c}-T_{t}^{g}+L_{T},t=1,\ldots ,T \end{array} \end{array}$$

Where, *CFF*_*t*_– net cash flow of the project in the framework of financial analysis in year *t*;

*I*_*t*_– investment in fixed and working capital in year *t*;

$X_{t}+T_{t}^{VA}$– clinics' income, including main revenue *X*_*t*_ and compensation for value-added tax in the operating period $T_{t}^{VA}$;

*M*_*t*_–operating expenses (including VAT, but without depreciation);

*T*_*t*_– tax payments in year *t*;

*L*_*T*_– terminal value in the last year *T*.^1^

For years of the duration of the PPP contract *t* = 1, …*T*_*p*_ the concessionaire is the owner of the objects being created, then for years *t* = *T*_*p*+1_…*T* the clinics become the owner. Accordingly, the system of cash flows differs for these periods. Let us consider a variant of the PPP project, considering three main taxes in years *t*: income tax $T_{t}^{i}$, property tax $T_{t}^{p}$and value-added tax. The latter is included in the capital and current costs, but the project participants do not pay it. However, compensation for value-added tax in the operating period is provided as part of the clinics' income. The income tax is partially paid by the concessionaire in the amount of $T_{t}^{ic}$, partially by clinics in the amount of $T_{t}^{ig}$ ($T_{t}^{i}=T_{t}^{ic}+$ $T_{t}^{ig}$). The owner pays the property tax, so in years *t* = 1, …*T*_*p*_ the concessionaire first transfers it to the budget, and later in years *t* = *T*_*p*+1_…*T* the clinics pay the tax. As a result, the budget receives the sum of two taxes in the amount of $T_{t}=T_{t}^{i}+T_{t}^{p}$.

As a result of the project, the construction of clinic buildings equipped with modern medical equipment is ensured. It is assumed that clinics are typical medical organizations of the public sector, which are budgetary institutions, the income of which is mainly formed at the expense of the state and local governments, as well as the funds of compulsory medical insurance. Such funds should be sufficient to cover the bulk of operating expenses. In addition, they are supplemented by partial receipt of income from the provision of services on a reimbursable basis, which leads to profit formation and is subject to income taxes. With the characteristics of a non-profit medical organization prevailing, the main cash flows of the project are in the form of substantial cash flows during the investment period and a significant cash inflow during the liquidation period. This feature of cash flow dynamics is typical for budgetary organizations. Hence, the model's financial bloc for such organizations includes the analysis of the return on investment in fixed and working capital, taking into account the terminal value. All indicators are calculated in the model in base and current prices.

As part of the financial analysis, the project is valued using a standard set of discounted efficiency indicators, primarily net present value *NPV*_*F*_ with the traditional financial discount rate *r*_*f*_.

The second group of cash flows is used in the economic bloc of the model to assess economic efficiency. These cash flows are determined by adding to the first group specific cash flows associated with special public benefits and costs, that are significant to society but difficult to observe as an outcome of the project, consequently, to measure them in monetary terms. For example, for health projects, such specific cash flows occur in the case of positive public effects resulting from expanding opportunities for quality treatment and subsequent recovery of patients. As a result, the economic bloc of the project model forms the basis for measuring in monetary terms social effects *S*_*t*_, tax effects *T*_*t*_, and other specific public effects *R*_*t*_ (environmental, price, indirect) in year *t*, which are presented in equation (2).


(2) $$\begin{array}{l} CFE_{t}=CFF_{t}+S_{t}+T_{t}+R_{t} \end{array}$$

In the current experimental calculations, we set *R*_*t*_ = 0.

### 2.2. Econometric methods for calculating social effects

For healthcare projects, the main focus in the transition from financial efficiency to economic efficiency is assessing social effects. In this paper, for this purpose, the method of fiscal multipliers is applied, with the value of operating expenses, excluding depreciation and VAT, being multiplied by a given multiplier at each point in time.

We employ the regression methodology for calculating the multipliers. Our data is panel data for 2005–2018 for 80 Russian regions. We apply fixed effects estimators that control for time invariants unobserved heterogeneity – any individual specific attributes that do not vary over time. We choose fixed effects specification based on the Hausman test. Fixed effects provide causal estimates in the absence of reverse causality.

We apply robust standard errors to control for heteroskedasticity and multicollinearity in our data. Robust standard errors are a technique to obtain unbiased standard errors of OLS coefficients under heteroskedasticity and multicollinearity. We cluster errors at the regions' level.

The data comes from “Regions of Russia” statistical yearbook (22), EMISS Federal statistics online service,^2^ and annual reports on human capital.^3^ We have three different multipliers. For the first multiplier, our dependent variable is GRP per capita. For the second multiplier, we have two regressions with GRP per capita and Human Development Index as dependent variables. Finally, for the third multiplier, the dependent variables are life expectancy and GRP per capita (see section 3 for details).

### 2.3. The efficiency of the project and the participation in the project

For the realization of a project, the problems of financing and corresponding redistribution of the results are crucial. The cash flows discussed above can be used to calculate relevant efficiency indicators, in particular, net present values within the framework of financial efficiency analysis:


(3) $$\begin{array}{l} \sum_{r=1}^{R}NPV_{r}^{F}=NPV^{F},\;\sum_{r=1}^{R}NPV_{r}^{E}=NPV^{E} \end{array}$$

Where $NPV_{r}^{F}$ is the financial net present value of the *r*-th participant of the project, which equals the sum of the discounted cash flow of the *r*-th participant *CFF*_*rt*_ and the financial net present value of the project *NPV*^*F*^ within the framework of financial analysis; *NPV*^*E*^ is the economic net present value of the project, which equals the sum of the economic net present values of the *r*-th participants and corresponding discounted cash flows *CEF*_*rt*_ within the framework of economic analysis. Formula 3 can include any project participants. In our case, these are government, concessionaire, commercial bank and clinic.

The efficiency indicators of participation in the project are presented on the left sides of the Equation (3), and the project efficiency indicators are on the right. Different mechanisms for project implementation led to corresponding ways of redistributing the total cash flows between the participants. However, the project's efficiency potential, the size of the “pie” created through its implementation, remains practically unchanged (except for tax liability adjustments for various financing schemes). This is true for both financial and economic analysis.

### 2.4. The relationship of cash flows in the public-private partnership project

Let's consider the relationship between the efficiency of the project and the efficiency of participation in the project on the example of the PPP mechanism for the construction of urban clinics initiated by the regional government and the corresponding relationship between the cash flows of its participants.

Let us renumber the project participants as follows. Government cash flows associated with the budgetary inflow and outflow are taken into account with number 1, combining traditional government participation in projects with budget revenues in the form of taxes and budget funding in the amount of $F_{t}^{1}$ in the period *t*. Following the typical contract structure for a PPP project, we single out the following three groups of participants with the corresponding numbers: concessionaire as a project company (2), commercial bank (3), and outpatient clinics (4). During the period of the PPP agreement, the cash flows of clinics are supplemented by the fulfillment of the obligations of the regional government for investment and operating payments. The concessionaire also acts as an investor and raises financing in the form of an equity loan in the amount of $F_{t}^{2}$ and a bank loan in the amount of $F_{t}^{3}$. The public partner transmits investment and operating payments to the concessionaire. The investment payment in the amount of *IP*_*t*_ as the part of cash inflows fully covers the corresponding expenses of the concessionaire for the repayments and the interest payments plus commission fees for both the equity loan in the amount of $IP_{t}^{3}(IP_{t}=IP_{t}^{2}+IP_{t}^{3})$. The operating payment consists of non-tax *OP*_*t*_ and tax $T_{t}^{p}\;$components. It is allocated to provide profitability to the concessionaire and to cover its operating expenses $M_{t}^{c}\;$and costs of paying property tax. The financial plan equations vary by periods of the duration of the PPP contract and subsequent years:


(4) $$\begin{array}{l} \begin{array}{lll} \Omega_{t}^{2} & = & {\sum^{\text{​}}}_{r=1}^{3}F_{t}^{r}+(IP_{t}+OP_{t}+T_{t}^{p})-(I_{t}+M_{t}^{c}+T_{t}^{p}+T_{t}^{ic}) \end{array}\\ \;\begin{array}{ll} - & IP_{t},\;t=1,\ldots ,T_{p}, \end{array} \end{array}$$

(5) $$\begin{array}{lll} \Omega_{t}^{4} & = & \left(X_{t}+T_{t}^{VA}\right)-\left(M_{t}+T_{t}\right)+L_{t},t=T_{p+1},\ldots ,T \end{array}$$

Where, $\Omega_{t}^{2}$ and $\Omega_{t}^{4}$ are the net cash flows for financial planning in the year *t* first of the concessionaire and then of the clinics. In our project, the financing is carried out by three participants so $F_{t}^{4}=0$ and is not part of formula (4).

During the period of the PPP contract, *t* = 1, …, *T*_*p*_, the cash flows of the four project participants can be represented as follows.


(6) $$\begin{array}{lll} CFF_{t}^{1} & = & {-F}_{t}^{1}+T_{t}^{p}+T_{t}^{ic}+T_{t}^{ig}-T_{t}^{VA} \end{array}$$

(7) $$\begin{array}{lll} CFF_{t}^{2} & = & \Omega_{t}^{2}+IP_{t}^{2}-F_{t}^{2} \end{array}$$

(8) $$\begin{array}{lll} CFF_{t}^{3} & = & IP_{t}^{3}-F_{t}^{3} \end{array}$$

(9) $$\begin{array}{lll} CFF_{t}^{4} & = & \left(X_{t}+T_{t}^{VA}\right)-\left(M_{t}^{g}+T_{t}^{ig}\right)-IP_{t}^{2}-IP_{t}^{3}-OP_{t}-T_{t}^{p} \end{array}$$

If we sum up the cash flows of all participants, presented in Equations (6–9), we will get the cash flow of the project, presented in Equation (1) and, consequently, the first part of the Equation (4) in terms of the relationship between financial efficiency of the project and participation in the project. This is explained by the presence of two identical amounts of benefits and costs accompanying mutual contracts for financial activities and tax interaction, which are mutually compensated by summation. A special role is played by the components of the financial plan, duplicating the components of the participants' cash flows, but with the opposite sign. Similar results are obtained within the framework of economic analysis. In this case, the cash flows of recipients of social effects are clearly shown as part of the flows to calculate the economic efficiency of participation in the project and the result of their summation to calculate the project's economic efficiency.

## 3. Results

### 3.1. Social effects multipliers

Below we present our calculations of the social effects multipliers.

In *Option 1*, in the first model, we regress GRP per capita on the public healthcare expenditure. Public healthcare expenditure is a sum of payments to TFOMS^4^ and the consolidated budget. Since we only have data on public health expenditure for 2011-2018, our regression is for this analysis period. Table 1 presents the results.

**Table 1:** Multiplier calculation (M1) in the fixed effects regression.

	**(1)**
**Variables**	**GRP per capita**
Public health expenditure	0.394^***^
	(0.099)
Constant	102,083.658^***^
	(3,033.761)
Observations	640
R-squared	0.067
Number of regions	80

Robust standard errors in parentheses. ^***^p < 0.01. Dependent variable GRP per capita, 2011–2018.

Our beta coefficient or multiplier M1 equals 0.39 and is statistically significant at the 1% significance level. We then multiply the expenditure from building the clinics by the multiplier and estimate the growth of GRP (gross regional product) per capita.

*Option 2* is a complicated relationship of the following form: dependence of the human capital on healthcare expenditure and dependence of GRP on the human capital. This estimation proceeds as follows. First, we multiply clinics' expenditures by the first multiplier (M2.1) and find the change in human capital. Then this change is multiplied by the second multiplier (M2.2) to yield the change in GRP per capita.

We proxy human capital by the human development index. It comprises life expectancy, income level, and education level. Since we only have human capital data from 2013, our analysis period is 2013–2018. Table 2 shows the estimation results.

**Table 2:** Calculation of M2 in the fixed effects regression, models 2 and 3, 2013–2018.

	**(2)**	**(3)**
**Variables**	**Human development index**	**GRP per capita**
Public health expenditure	2.38 e^−7***^	
	(0.000)	
Human development index		619,550.489^***^
		(94,503.444)
Constant	0.817^***^	−393,989.854^***^
	(0.002)	(77,910.018)
Observations	480	480
R-squared	0.023	0.255
Number of regions	80	80

Robust standard errors in parentheses. ^***^ p < 0.01.

From the model that relates the human development index to PHE, we get M2.1 = 2.38 e^−7^. The multiplier is very small because health expenditures are in mln rubles, and the human development index is bounded between 0 and 1. We find M2.2 from the model (3). M2.2 = 619550.49. The composite multiplier for 1 and 2 is the multiplication of the two and equals M2 = 0.147.

*Option 3* is another composite multiplier (M3) which consists of the two multipliers (M3.1 and M3.2). We have a figure for an increase in the public health expenditure from the project of opening the clinics, and we multiply it by M3.1 to get a figure of life years gained (Model 4). Multiplication of the life years gained by M3.2 (Model 5) produces an increase in GRP per capita. This is the estimate of the social effect of the project. Results are presented in Table 3.

**Table 3:** Calculation of M3 in the fixed effects regression, models 4 and 5, 2013–2018.

	**(4)**	**(5)**
**Variables**	**Life expectancy**	**GRP per capita**
Public health expenditure	0.000045^***^	
	(0.000)	
Life expectancy		5,219.729^***^
		(861.985)
Constant	69.126^***^	−253,893.813^***^
	(0.344)	(60,784.835)
Observations	640	640
R-squared	0.101	0.239
Number of regions	80	80

Robust standard errors in parentheses. ^***^p < 0.01.

M3.1 = 0.000045; M3.2 = 5219.73. The total multiplier for Option 3 is M3 = 0.245.

In all previous options, multipliers lie between zero and one. For the following two options, we applied an empirical methodology of the budget multiplier method based on the Center for Strategic Research (23) results and approach to Structural Vector Autoregression (SVAR) estimation. This is the modification of the classical method of vector autoregression (VAR) and differs from the latter in that it imposes additional restrictions on the coefficient matrices. The experts used Rosstat data on Russia's GDP from 2000 to 2016 at current prices and constant 2008 prices (in quarterly terms), as well as data from the Federal Treasury on the execution of the budget of the enlarged government of the Russian Federation for 2000–2016. It is shown that the coefficients are statistically significant at the level of 10%. We borrowed the estimates for *options 4 and 5* from the Center of Strategical Research method.

As an intermediate, we considered *option 4*, in which the budget multiplier was built for the total budget spending in Russia in the period and was equal to 0.91.

As the main one, we consider *option 5*, which uses the multiplier for Russian health care spending, which is equal to 1.25. It should be taken into account that the range of multiplier values obtained in this study is quite broad. For example, for spending on road infrastructure and transport, the multiplier was 1.64. Considering the spread of multipliers for other estimates, option 5 for the base case seems to be justified.

### 3.2. Financial and economic efficiency of the project

The considered methods were tested on the example of a project to construct seven polyclinics to provide primary health care in Novosibirsk. In 2019, a PPP Agreement was signed between the government of the Novosibirsk Region and the Seventh Concession Company on constructing new buildings for existing polyclinics on allocated land plots. The company is a member of the VIS group, a Russian holding implementing infrastructure projects and one of the leaders in the PPP market. The project involves not only the planning and construction of buildings but also the provision of equipment, including purchasing complexes for computed tomography, MRI, mammalogical systems, ultrasound, and X-ray units. Their total capacity is expected to be almost 6,500 visits per shift. At the time of the project launch, the project implementation period was set at ten years, and the cost of building and equipping clinics was estimated at 7.8 billion rubles (USD 112.3 mln)^5^ in current prices ♯ 6.4 billion rubles (USD 92.1 mln) in constant prices of 2018. Subsequently, the project cost was increased, first to 9.7 billion rubles (USD 139.6 mln) in 2021, then 19.5 billion rubles (USD 280.7 mln) in 2022, with adjustments to the schedule for commissioning facilities and the completion date of the project. Experimental calculations for the project were carried out according to the initial version of the 2019 Agreement.

The main results of the evaluation of the efficiency of the project under consideration are given in Table 4.

**Table 4:** The main efficiency indicators for the outpatient clinics' project for period 2019–2035.

	**NPV, thousand RUR/thousand USD**		**IRR, %**	**Payback period, years**
	**(*****r** =* **0%)**	**(*****r** =* **7.3%)**		
Financial efficiency	1 396 478 (USD 20 102)	−1 741 602 (USD −25 070)	2.17%	16
Budgetary efficiency of the project	−2 190 802 (USD−1 536)	−2 722 898 (USD −39 195)	–	–
Economic efficiency of the project	30 726 092 (USD 442 293)	12 966 953 (USD 186 655)	41.36%	6

All performance indicators show the unconditional interest of society in implementing the project. Such impressive results arise primarily due to social effects (Table 5), amounting to 193 139 thousand dollars and providing the main contribution to the economic NPV of the project in the amount of 103.5% (taking into account the negative value of the contribution of financial efficiency).

**Table 5:** The main effect for the outpatient clinics' project for the period 2019–2035.

	**Total amount, thousand RUR/thousand USD**		**Structure, %**	
	**Without discounting**	**With discounting**	**Without discounting**	**With discounting**
	**(*****r** =* **0%)**	**(*****r** =* **7.3%)**	**(*****r** =* **0%)**	**(*****r** =* **7.3%)**
Financial efficiency	1 396 478 (USD 20 102)	−1 741 602 (USD−25 070)	4.54%	−13.43%
Tax effects	1 991 796 (USD 28 671)	1 291 213 (USD 18 587)	6.48%	9.96%
Social effects	27 337 818 (USD 393 520)	13 417 343. (USD 193 139)	88.97%	103.47%
Economic efficiency	30 726 092 (USD 442 293)	12 966 953 (USD 186 655)	100.00%	100.00%

### 3.3. Economic efficiency of the project with various options for assessing social effects

The methods discussed for assessing social effects were utilized to calculate the corresponding options for the project of polyclinics, presented in Table 6. They served as the basis for constructing cash flows for the transition from the same initial version of financial analysis to the appropriate options of economic analysis. For measurement, the methods of NPV of cash flows for social effects and economic NPV of the project are applied. The results obtained regarding the value of social effects differ by 3.2 times (from the lowest in option 1 to the highest in option 5), with the results on NPV of economic efficiency (EE) differing by 2.5 times without discounting while 3.4 times with discounting.

**Table 6:** Social effects and economic efficiency (EE) for the outpatient clinics' project for the period 2019–2035.

	**Social effects**,		**Economic efficiency**,		**Social effects**,	
	**thousand RUR/thousand USD**		**thousand RUR/thousand USD**		**% of EE**	
	***d** =* **0%**	***d** =* **7.3%**	***d** =* **0%**	***d** =* **7.3%**	***d** =* **0%**	***d** =* **7.3%**
Option 1	8 529 399 (USD 122 778)	4 186 211 (USD 60 259)	12 088 371 (USD 174 009)	3 754 697 (USD 54 048)	70.6%	111.5%
Option 2	3 214 927 (USD 46 278)	1 577 880 (USD 22 713)	6 773 899 (USD 97 508)	1 146 366 (USD 16 502)	47.5%	137.6%
Option 3	5 358 212 (USD 77 130)	2 629 799 (USD 37 855)	8 917 184 (USD 128 360)	2 198 286 (USD 31 644)	60.1%	119.6%
Option 4	19 901 931 (USD 286 428)	9 767 826 (USD 140 605)	23 460 903 (USD 337 713)	9 336 312 (USD 134 393)	84.8%	104.6%
Option 5	27 337 818 (USD 393 520)	13 417 343 (USD 193 139)	30 514 293 (USD 439 244)	12 748 832 (USD 183 516)	89.6%	105.2%
Ratio 5 to 1	3.2	3.2	2.5	3.4	1.3	0.9

### 3.4. The efficiency of participation in the outpatient clinics' project

The proposed model is invisibly present “behind the scenes” of all PPP projects, making it possible to identify those potential net benefits - the size of the future “pie” that is redistributed among the participants in investment activities under various implementation mechanisms. The size of this “pie” is determined using the most straightforward mechanism of project financing – entirely from its capital, in this context, from the regional budget of the Novosibirsk region.

Table 7 shows the distribution of the project's net present value among its participants in the framework of financial and economic analysis (at various discount rates). For the direct participants of the project, both private and public partners, the results are relatively low, in contrast to the economic NPV and the corresponding consistently high share of the efficiency for the residents of the Novosibirsk region and, above all, patients of the established polyclinics.

**Table 7:** The relationship between the efficiency of the project and participation in the project.

	**Thousand rubles**		**% of EE**	
**Net present value**	**(*****r** =* **0%)**	**(*****r** =* **7.3%)**	**(*****r** =* **0%)**	**(*****r** =* **7.3%)**
Financial efficiency of the project	1 396 478	−1 741 602	4.5%	−13.4%
NPV for concessionaire	1 002 350	395 617	3.3%	3.1%
NPV for bank capital	2 317 716	489 515	7.5%	3.8%
Total efficiency for private participants	3 320 066	885 132	10.8%	6.8%
NPV for clinics	−601 452	−1 558 389	−2.0%	−12.0%
Total efficiency for micro-level participants	1 396 478	−1 741 602	4.5%	−13.4%
Budgetary efficiency	−1 589 350	−1 164 510	−5.2%	−9.0%
Efficiency for the public partners	−2 190 802	−2 722 898	−7.1%	−21.0%
Economic efficiency of the project	30 726 092	12 966 953	100.0%	100.0%
Efficiency for residents of the region	27 337 818	13 417 343	89.0%	103.5%
Efficiency of participation in the project for all participants	30 726 092	12 966 953	100.0%	100.0%

## 4. Discussion

The project performance indicator system significantly depends on using cash flow discounting methods. When calculated using simple non-discount methods, the project provides a positive financial net present value (NPV) of 20 102 thousand dollars. For discounting, we applied the 7.3% rate, calculated and applied by the developers of the PPP project. Using discounting methods, it becomes clear that the project is financially inefficient. The internal rate of return of the project is 2.17% (Table 4). The project generates a net NPV loss of −25 070 thousand dollars, while the obtained internal rate of return (IRR) is below the accepted discount rate, and the payback period practically coincides with the project's duration.

However, the conclusions fundamentally change in the transition to the economic efficiency assessment of the project, taking into account its social and tax effects. Discounted methods indicated the exceptionally high performance of the project. The project's economic NPV at a 7.3% discount rate reaches 186 655 thousand dollars in the economic efficiency assessment. Moreover, the IRR of 41.36% (Table 4) is significantly higher than the accepted discount rate, indicating a significant margin of safety concerning possible adverse changes. The project pays off, according to the results of calculations using the discounting method, in 6 years.

It is also necessary to notice the high share of terminal (liquidation) value in the total value of the project's performance indicators in both financial and economic analysis. The value, estimated during the conditional completion of the project in 2035, indicates a high potential for further development of outpatient clinics. This is typical for social infrastructure projects in general, and in particular for healthcare projects focused on providing free medical services. The corresponding contribution of the liquidation value to the economic NPV of the project is equal to 6.5% when estimating without discounting and 4.7% with discounting.

For the evaluation of PPP projects, a clear separation of the efficiency analysis of the project and participation in the project is of particular importance. Most often, the efficiency of the concessionaire's participation in the project is presented as project performance indicators that are highly objectionable for two reasons. First, it is essential to distinguish between the evaluation of the project and the consequences of its implementation using different mechanisms (including various forms of government support). Secondly, this approach focuses on only one participant, the concessionaire. At best, the project is also presented from the perspective of a public partner, but only in the part of investment and operating payments as the main components of the concession fee. In this case, the public partner is again considered only as a counterparty to the PPP agreement, providing the income for the concessionaire.

Meanwhile, a critical participant in PPP projects is the bank, which is generally not specified in PPP agreements. Yet, debt service obligations on a bank loan are included in the investment payment provided by the concessionaire. In the results of the efficiency of participation in the project, it is essential to highlight the shares of all participants, particularly the share of the bank as one of the most important private agents in real PPP contacts. As demonstrated by calculations, in all examined options for the implementation of the project, bank capital provides a stable high share, which is associated with a noticeable excess of the interest rate on loan (10.5%) compared to the discount rate. The share of the concessionaire varies significantly with different methods of state support for the project. In the base case, it was supposed to provide the concessionaire with a tax benefit in the form of an exemption from paying income tax (often provided for socially significant PPP projects in Russia).

Consider how different options for government tax support affect the situation of project participants. In the first version, with the refusal to provide the concessionaire with benefits in the form of exemption from income tax, its net cash flow for financial planning will have a steady negative value in the operating period, which leads to a low level of financial NPV for concessionaire of 48.7 thousand dollars, or 0.5% of the economic NPV. Bank capital still receives its positive NPV at the same level as in the base option.

In the additional version, with additional refusal to compensate the property tax payments in the operating payment for the concessionaire, the discounted financial NPV for the concessionaire becomes negative, amounting to −1 155 thousand dollars, or −0.6% to the economic NPV. Bank still receives its steady positive NPV of USD 7 046 thousand dollars or 3.8% of the economic NPV.

However, the bulk of the beneficial effects of the projects still accounts for the share of regional residents, for whom the discounted NPV is 193 139 thousand dollars or 103.5% of the economic one.

## 5. Conclusion

Our study presents a cost-benefit analysis of the project for constructing seven polyclinics in the Novosibirsk region of Russia. Along with financial and economic efficiency, we aim to estimate the social efficiency of the project as gains for clients of the polyclinics. We choose CBA as the most complete and systematic methodological tool for evaluating social infrastructure projects in monetary terms.

The proposed methods for evaluating a PPP project make it possible to quantify the income distribution from the project implementation between its participants. The concessionaire's cash flow differs significantly from the corresponding project cash flow figures. The concessionaire's corresponding NPV at 7.3% is only 3.1% of the project's economic NPV with the project's negative financial NPV. The proposed approach reveals a stable high positive share of the bank in the overall results, consistently at the level of at least 3.8%.

The project's primary outcome is the accumulation of health capital of the “polyclinics” patients and residents of the region. As a direct result of the project implementation, “polyclinics” patients have the opportunity to receive new medical services. This is very important given the current shortages of medical personnel. To estimate the effect of health capital accumulation in monetary terms, we applied CBA methodology of budgetary social multipliers. We estimate the multipliers *via* the econometric framework of the fixed effects regression based on the data for Russian regions. The multipliers varied between 0.147 and 1.5, and the discounted social effects' estimation ranged between 22 713 and 193 139 thousand dollars. We believe that positive social effects indicate a successful project that can set an example in the region's health care for other new public health PPP projects.

## Data availability statement

The raw data supporting the conclusions of this article will be made available by the authors, without undue reservation.

## Author contributions

TN conceived and designed the study. TN and MK developed the research model. MZ conducted data collection and analysis. MK performed the statistical analysis. MZ and MK drafted the Introduction section, while TN drafted the rest of the manuscript. All authors contributed to the manuscript revision, read, and approved the submitted version.

## Footnotes

^1^All variables of cash flow in formula 1, except for taxes, are defined by experts based on the initial information about the project. For our projects of seven polyclinics these data were extracted from the PPP contract dated 2018. Tax variables were calculated based on the parameters of the tax system of the Russian Federation for the beginning of 2019.

^2^EMISS government statistics [Internet]. Available from: https://fedstat.ru/.

^3^Map of the subjects of the Russian Federation and their Human Development Indexes [Internet]. 2017. Available from: https://ac.gov.ru/uploads/2-Publications/karta_HDI_2017.pdf.

^4^Territorial Fund of Compulsory Medical Insurance – one of non-budget funds through which payments for treatment of the Russian citizens are made.

^5^The rate of 69.47 RUR for one USD for January 2019 is used in the paper for conversion of rubles into dollars.
